# Evolutionary phylogeography and transmission pattern of echovirus 14: an exploration of spatiotemporal dynamics based on the 26-year acute flaccid paralysis surveillance in Shandong, China

**DOI:** 10.1186/s12864-016-3418-3

**Published:** 2017-01-07

**Authors:** Peng Chen, Yan Li, Zexin Tao, Haiyan Wang, Xiaojuan Lin, Yao Liu, Suting Wang, Nan Zhou, Pei Wang, Aiqiang Xu

**Affiliations:** 1 Department of Epidemiology, School of Public Health, Shandong University, No. 44 Wenhuaxi Road, Jinan, 250012 People’s Republic of China; 2 Shandong Provincial Key Laboratory of Infectious Disease Control and Prevention; Shandong Center for Disease Control and Prevention, No. 16992 Jingshi Road, Jinan, 250014 People’s Republic of China

**Keywords:** Echovirus 14, Acute flaccid paralysis, Phylogeny, Phylogeography

## Abstract

**Background:**

Echovirus 14 (E-14) causes various clinical recognized syndromes, mostly with gastrointestinal syndrome and paralysis. The current study summarized the Shandong E-14 strains isolated from a 26-year acute flaccid paralysis (AFP) surveillance, and elucidated the characterization of phylogenetic and phylogeographic relationships of E-14 worldwide.

**Results:**

As a predominant serotype circulating in AFP surveillance, phylogenetic analysis showed that E-14 exhibited both time and geographic subdivision worldwide. In order to know the evolutionary history and spatial temporal dynamics of E-14, evolutionary phylogeography was reconstructed using BEAST and SPREAD software based on the VP1 sequences. The time of the most recent common ancestor of E-14 was estimated around 85 years and evolved with 9.17 × 10^−3^ substitutions/site/year. Phylogeographic analysis suggested that two regional transmissions of E-14 were mainly detected, with one located between Europe and Africa countries and the other was in the Asia-Pacific region.

**Conclusions:**

Our study investigates the molecular evolution and phylogeographic of E-14, and brings new insight to the dispersal of E-14 worldwide. Regional transmission was mainly detected and Australia may be responsible for the spread of E-14 in recent years.

**Electronic supplementary material:**

The online version of this article (doi:10.1186/s12864-016-3418-3) contains supplementary material, which is available to authorized users.

## Background

Enteroviruses (EVs) are common human pathogens belonging to the genus *Enterovirus*, family *Picornaviridae*. Most EV infections are usually asymptomatic, but sometimes they are associated with diverse clinical syndromes ranging from minor febrile illness to severe, potentially fatal diseases such as encephalitis, paralysis, myocarditis and neonatal enteroviral sepsis [1, 2]. EVs have traditionally been detected based on virus isolation in cell cultures and subsequently serotyped by serum neutralization test, and were classified into echoviruses (E), coxsackieviruses (CV) group A and B, and polioviruses. Currently, based on the phylogenetic relationships in multiple genome regions, International Committee on Taxonomy of Viruses reclassified EVs into 12 species: EV-A to EV-H, EV-J and rhinovirus A, B and C [3]. Of these species, seven (EV-A to EV-D and rhinovirus A, B, C) of the EV species infect humans while remaining infect non-human animals.

Echovirus 14 (E-14) was first isolated from a hospitalized patient suffering from aseptic meningitis syndrome in 1954 [4] and had been classified as a member of EV-B. In contrast to other common EVs such as EV-A71 and CV-A16, both of which are categorized as EV-A and are major etiological agents for the widely spread hand, foot and mouth disease (HFMD) [5–7], E-14 is considered relatively rare as it less likely to cause worldwide occurrences of clusters and outbreaks. However, epidemics of E-14 infection had been observed in Europe and Asia in the 1960s [8–11]. Similarly, association of E-14 with many clinical recognized syndromes such as gastrointestinal syndrome [8–10], acute serous meningitis [12], paralysis [13], fatal neonatal hepatic necrosis [14], aseptic meningitis [11, 15] has been reported previously. In recent years, E-14 is more than detected in an outbreak or epidemic caused by other pathogens [16, 17] or exhibited sporadic infection in conventional virological surveillance [18–20].

As a coastal province, Shandong has a population of about 96 million and major ports that could potentially serve as portals for importation of exogenous viruses. The acute flaccid paralysis (AFP) surveillance, developed for polio eradication program, was launched in Shandong in 1988. Currently, the AFP surveillance has been conducted in all 138 counties of Shandong province and more than 600 sentinel hospitals were included. AFP surveillance can obtain non-polio enterovirus (NPEV) strains as a side benefit and produce baseline data of local NPEV distribution and a genetic overview [21]. Our previous study demonstrated continuous AFP surveillance provided valuable information on the circulation and emergence of different EV types in the context of limited EV surveillance in China [20]. In this present study, we investigated the genetic characterization of E-14 strains isolated from Shandong AFP surveillance during 1988–2013. Phylogenetic and phylogeographic analysis were combined to elucidate the evolution relationship and spatiotemporal dynamics of E-14 worldwide.

## Results

### Virus isolation

**Fig. 1:**
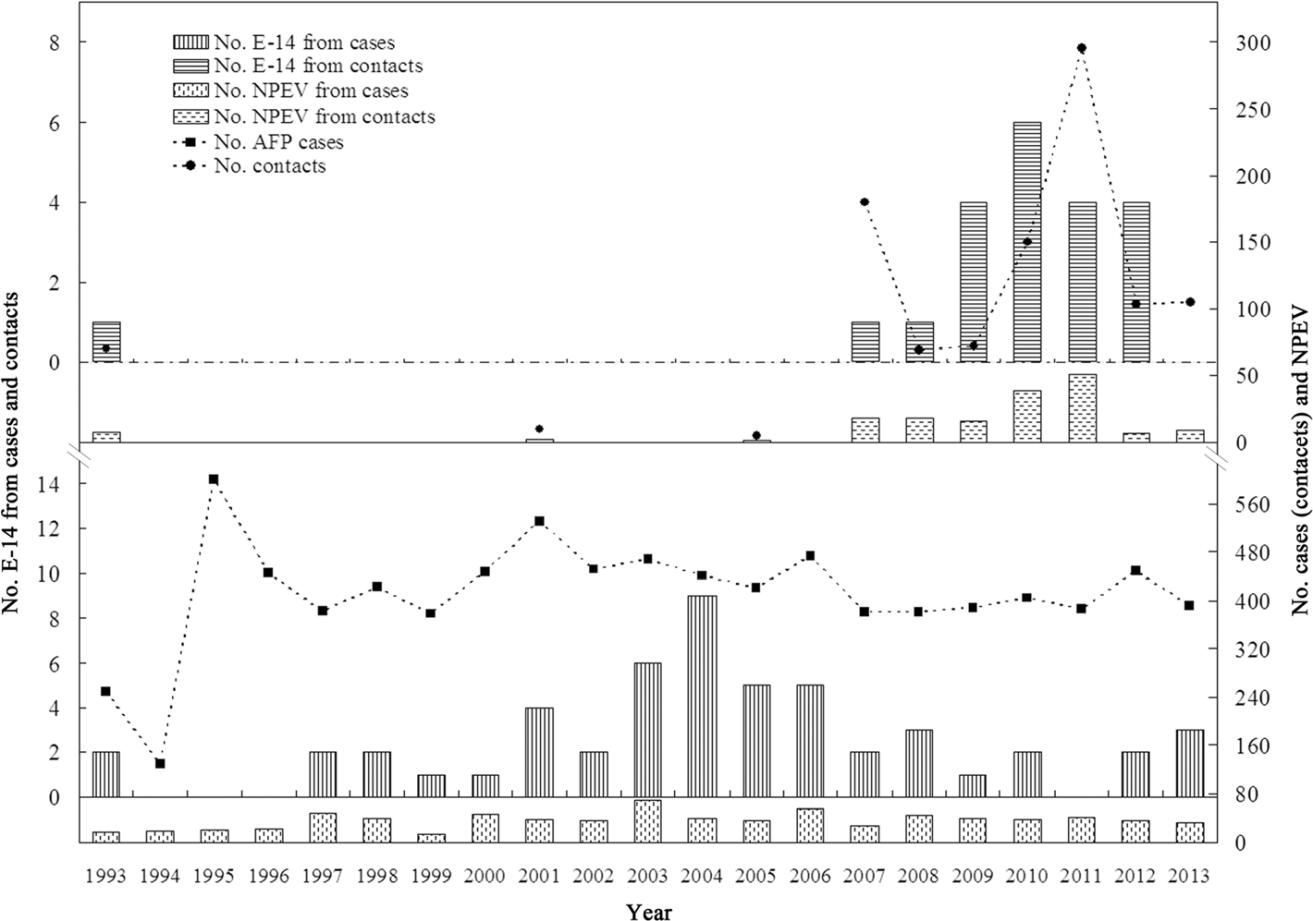
Annual numbers of AFP cases (contacts), NPEV and E-14 isolates in Shandong AFP surveillance from 1988 to 2013

The annual numbers of reported AFP cases (contacts), NPEV and E-14 isolates were illustrated in Fig. 1. From 1988 to 2013, a total of 962 NPEV isolates were identified based on the molecular typing method in Shandong AFP surveillance, with E-14 serotype having the highest number of the total strains. In our study, E-14 was first detected in 1993. As the AFP surveillance and report in China was not so active in the early 1990s, no E-14 isolates were detected for three consecutive years (1994–1996), and numbers of E-14 and NPEV strains increased substantially since 1997. It was worth noting that the proportion of E-14 in contacts is higher than in AFP cases is due to the formation of EV transmission chains, for the rest contacts were probable positive as one infected E-14. The mean constituent rate of E-14 was 7.6% (73/962), and the highest constituent rate reached to 23.1% (9/39) in 2004. Consequently, E-14 under EV-B persisted to be the most commonly isolated serotype circulating in a long term trend in our AFP surveillance.

### Homology and phylogenetic analysis

**Fig. 2:**
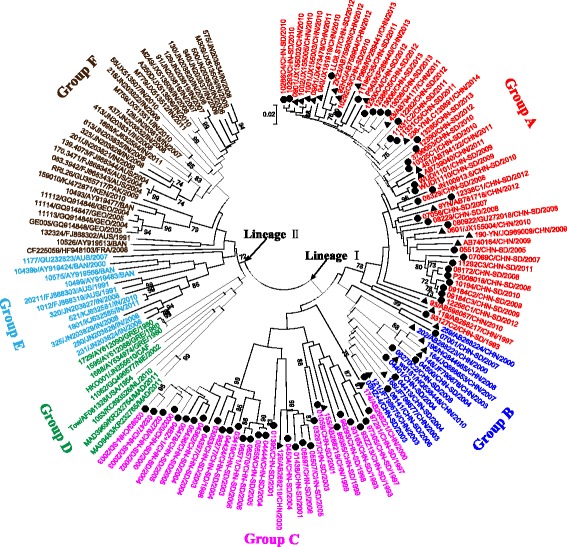
Phylogenetic tree based on partial E-14 VP1 sequences of Shandong isolates and global strains. Strains are color-coded and represented group A-F according to their phylogenetic relationship. Circles indicate strains detected in the present study; triangles indicate strains isolated from other provinces in China. Abbreviations in isolate names are as follows: CHN-SD, Shandong province in China; USA, United States of America; AUS, Australia; IN, India; FRA, France; BAN, Bangladesh; MAD, Madagascar; NL, Netherlands; NIE, Nigeria; CAF, Central African Republic; GRE, Greece; GEO, Georgia; KEN, Kenya; PAK, Pakistan

Excluding the same (100% VP1 nucleotide homology) strains and co-infection strain, we used 73 E-14 strains isolated from Shandong province for phylogenetic analysis based on the VP1 sequence. Homologous comparison showed 73 Shandong E-14 isolates had 79.8–99.8% (94.9–100% amino acid sequence identities) VP1 nucleotide sequence identities among themselves and the overall mean *p*-distance value was 0.142. Phylogenetic analysis was conducted on partial VP1 coding region (nt 2596–2880 on prototype strain Tow) of Shandong isolates with global reference E-14 sequences. Overall, the global E-14 sequences were distributed into two major partitions identified by lineage I and II. Compared with the global E-14 strains, all Shandong E-14 isolates fell into a major phylogenetic lineage (lineage I) that included strains from China (Fig. 2). This is probably due to the more frequent population exchange in domestic than the rest of the world, as the international exchange needs to perform a more cumbersome procedure in China. Therefore, isolates from China studied here existed distinct genetic relationship with other worldwide E-14 strains. We also suggested that E-14 strains from China had the same putative ancestor relative to the strains from other regions of the world.

Within lineage I, all sequences segregated into three distinct genomic groups (A-C). Homologous analysis revealed 79.9–88.7, 80.8–84.7, 79.8–86.3% nucleotide identities between groups A and B, A and C, B and C, respectively. Strains in these three groups showed high correlation with the time of isolation, and a limited temporal overlap was observed among these three groups that circulated in Shandong and other provinces in China. The phylogenetic analysis showed that strains in lineage II mainly from India, Australia, Georgia, Netherlands, France and Bangladesh. For the 26 India strains isolated from 2007 to 2011, 20 strains segregated into group F with strains from Australia, while the other 6 strains isolated from 2008 to 2011 belonged to group E in the phylogenetic tree.

### Phylogeographic analysis

In order to estimate the divergence time and substitution rate of E-14 more accurately, the complete VP1 sequence from Shandong province and from global were selected to analyze with the Bayesian MCMC method. Different models were used for data analysis, and all of the results were summarized in Table 1. Considering both the Harmonic Mean and the AICM model selection results, we concluded that the UCLD relaxed clock model was a significantly better fit model than the strict clock model for each of the parameter. Hence, the combination of UCLD relaxed clock model and exponential growth (EG) model were recommended for this study, and the estimated nucleotide substitution rate for global E-14 was 9.17 × 10^−3^ substitutions/site/year (95% HPD: 7.67 × 10^−3^–10.80 × 10^−3^). Estimated the *t*_*MRCA*_ of global E-14 strains was August 1928 (95% HPD: February 1910–May 1944), China strains (lineage 1) was June 1972 (95% HPD: February 1965–June 1979), and the strains isolated in other regions of the world (lineage 2) was September 1967 (95% HPD: January 1958–August 1976).

**Table 1:** Estimators of phylogeographic analysis on E-14 strains under different molecular clock and coalescent model combinations

Parameter	Estimator of the parameter (95% HPD interval) under different molecular combinations					
	SC & Const	SC & Exp	SC & BSP	UCLD & Log	UCLD & Exp	UCLD & BSP
Harmonic Mean	−18271.839	−18253.871	−20602.113	−18187.626	**−18169.821**	−20529.023
AICM	36747.72	36686.69	41416.886	36765.743	**36742.314**	41489.35
*t*_MRCA_ (global E-14)	88.06 (78.14,98.49)	87.74 (77.86,97.49)	86.29 (77.15,96.05)	85.48 (70.22,103.77)	**85.31 (69.64,103.79)**	83.02 (68.20,100.25)
*t*_MRCA_ (Lineage 1)	43.44 (39.22,47.96)	43.52 (39.36,48.18)	42.64 (38.81,46.97)	41.26 (34.69,48.50)	**41.46 (34.45,48.79)**	40.80 (34.92,47.36)
*t*_MRCA_ (Lineage 2)	46.87 (41.54,52.31)	47.04 (41.77,52.62)	45.92 (41.07,51.20)	46.32 (37.09,56.15)	**46.15 (37.33,55.94)**	44.41 (36.14,53.59)
*t*_MRCA_ (group 1)	32.31 (28.95,35.75)	32.36 (29.09,35.90)	31.73 (28.66,34.97)	31.50 (26.51,37.04)	**31.46 (26.35,36.96)**	31.38 (26.61,36.66)
*t*_MRCA_ (group 2)	29.85 (26.57,33.27)	29.89 (26.70,33.38)	29.45 (26.36,32.67)	27.41 (22.47,32.85)	**27.40 (22.49,32.77)**	28.09 (23.16,33.32)
*t*_MRCA_ (group 3)	29.63 (27.53,31.72)	29.70 (27.70,31.97)	29.53 (27.58,31.60)	28.45 (25.42,31.64)	**28.46 (25.26,31.61)**	28.85 (25.95,32.21)
*t*_MRCA_ (group 4)	46.43 (40.44,52.43)	46.62 (40.92,52.85)	45.63 (40.19,51.24)	45.47 (36.29,56.13)	**45.30 (35.46,55.05)**	43.72 (35.19,53.39)
*t*_MRCA_ (group 5)	38.91 (33.45,44.89)	39.05 (33.61,44.99)	38.33 (33.06,43.68)	34.62 (27.34,43.15)	**34.43 (27.34,42.15)**	33.55 (27.35,41.16)
Mean evolutionary rate	8.44 (7.42,9.48)	8.38 (7.39,9.46)	8.57 (7.61,9.58)	9.14 (7.65,10.70)	**9.17 (7.67,10.80)**	9.30 (7.80,10.80)

The best fitting model combination was highlighted in boldface. The molecular clock models used were the strict molecular clock (SC) and the uncorrelated log-normal distributed (UCLD) relaxed molecular clock. Coalescent models used were the parametric Constant model (Const), Exponential model (Exp), Logistic model (Log) and Bayesian skyline plot model (BSP). *95% HPD* 95% highest posterior density, *t*_*MRCA*_ the most recent common ancestor, *AICM* the Akaike information content model selection method

**Fig. 3:**
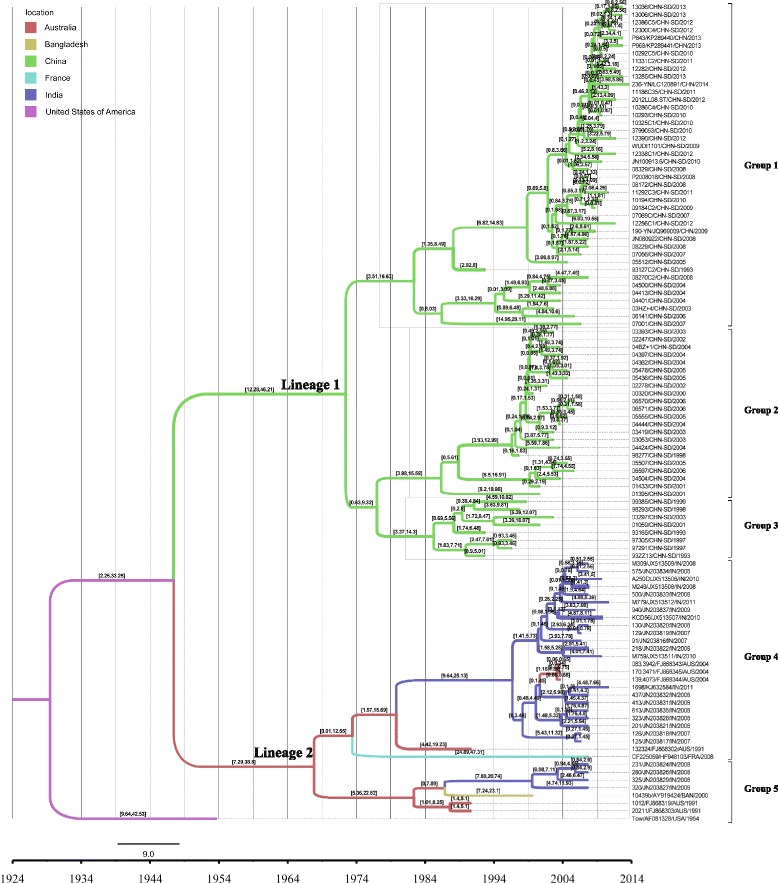
Maximum clade credibility (MCC) tree of the complete VP1 sequences of E-14 strains throughout the world visualized in FigTree. The colors of the branches corresponded to their probable geographic location. The intervals of branch reflect the 95% highest posterior density (HPD) intervals for the branch height. E-14 isolates from Shandong segregated into three groups (1, 2 and 3). Abbreviations of geographic location are shown as described in the legend of Fig. 2

Results of the MCC tree (Fig. 3) constructed by Tree Annotator showed that Shandong E-14 isolates segregated into three groups, which were consist with the phylogenetic tree in Fig. 2. Phylogenetic analysis suggested the origin of global E-14 could be in the USA. Lineage 1 represented strains from China (including all Shandong isolates), and the data indicated that E-14 in China shared the same putative ancestor. Lineage 2 represented the strains from other regions of the world, and the strains were gradually radiated into France, India and Bangladesh. Our results showed that strains in Australia may have also played a dominant role in spreading this lineage.

**Fig. 4:**
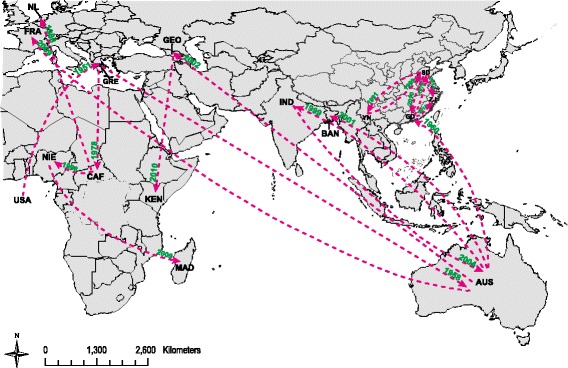
Geographic distribution and inferred dynamics of global E-14 strains. The map was reconstructed using the ArcGIS (http://www.esri.com/), and was identical to the original image created by the SPREAD and Google^TM^ Earth. Arrows indicate the inferred routes of spread of global E-14. The number represented the time when E-14 arrived. Abbreviations of geographic location are shown as described in the legend of Fig. 2

The geographic dispersal of the global E-14 strains was conducted with the partial VP1 sequences and was shown in Fig. 4 (Additional files 1 and 2). Phylogeographic reconstruction was able to identify a single location for the origin of the global E-14 strains in the USA around March 1928. The virus crossed the Atlantic Ocean and arrived in Greece in 1951, and then spread following two distinct routes: i) to the East arrived in Australia around 1958 and ii) to the South arrived in Central African Republic around November 1978. As E-14 strains arrived in Australia, the virus was latent in this region and spread to India, Georgia, Bangladesh and France in about 40 years. Viral strains that from Australia arrived in Guangdong province of China in an early time, and then showed a relatively quick diffusion rate to Shandong and Zhejiang provinces. In general, E-14 strains from the USA and Australia were the most probable source for the introduction of E-14 strains to European countries and the Asia-Pacific region, respectively, with all BF > 19.0.

## Discussion

Compared with many EV serotypes which can lead to serious illness, E-14 is considered relatively rare as it less likely to cause worldwide occurrences of clusters or outbreaks. To the best of our knowledge, no detailed molecular epidemiology study was carried out based on a large number of E-14 sequences. Owning to the launch of the Global Poliomyelitis Eradication Initiative, we had accumulated a significant number of E-14 sequences associated with AFP surveillance which was developed for casting a wider net for poliovirus detection in Shandong since 1988. For the detection of circulating EVs, AFP surveillance has also provided a major source of information in China, as specialized EV surveillance based on other approaches or populations was still very limited. In addition, studies conducted in other countries [18, 22–27] also described the NPEV serotypes identified from the AFP surveillance, and the information had attracted more attention of the scholars worldwide.

Although E-14 may cause mild to severe neurological disorders as reported from Russia [12], France [9, 10], Italy [8] and Japan [11, 13, 15], unfortunately, no clinical information, molecular epidemiology and disease correlation of this serotype have been reported. This study, which was performed a comprehensive molecular epidemiologic analysis of Shandong E-14 isolates, was feasible because of the large number of AFP database during the last 26 years in Shandong, China. During 1990–2013, a total of 10322 AFP cases and contacts were reported, and 958 NPEV isolates were obtained from stool specimens in Shandong AFP surveillance [20]. A total of 53 NPEV serotypes were identified, serotypes such as E-6, E-14 and EV-A71 were the most commonly reported. It should also be notified that no EV-D68 strains were detected in our AFP surveillance, although this EV serotype was associated with AFP and other neurological complications. The proportion of E-14 isolated in this study was high (7.6%) as compared to other identified NPEV serotypes [20]. Additionally, E-14 was also found a prevalent EV serotype with AFP surveillance in other regions of the world [18, 19, 28]. Consequently, all these findings suggest E-14 as the possible causative agent of paralysis, and the molecular epidemiologic analysis of E-14 is still necessary.

EVs are well known for their high genome plasticity, due to both high mutation and recombination rates. In this study, we analyzed the global E-14 genetic diversity, of 73 isolates from Shandong and 54 strains isolated in twelve different countries. The phylogenetic tree (Fig. 2) revealed that E-14 strains clustered in both time and regional distributions. For E-14 isolated in China, strains in group C and B were from 1993 to 2006 and 2000 to 2010, respectively and there were 10 and 6 years had passed since the last isolation in these two groups. The isolation years of group A spanned from 1993 to 2014, revealing a long time circulation of this group in Shandong region. Moreover, it is obvious that E-14 isolates belonging to group B and C may have been disappeared after circulating for a period of time and was replaced by group A. Viruses in group A may have become the dominant group circulating in China. Alternatively, the information on Chinese E-14 isolates is limited, and more sequences are needed to determine domestic E-14 phylogenetic relationships.

We used a phylogenetic molecular clock approach to further infer the historical background and geographic diffusion of global E-14 strains. The MCC tree (Fig. 3) constructed was congruent with the results observed in phylogenetic analysis (Fig. 2). It was perceived by Bayesian MCMC evolutionary analysis that E-14 strains were circulated for about 80 years in the world. Moreover, E-14 strains isolated in China may be the product of geographic segregation and has evolved independently in China for a long time. Two observations suggest that the common ancestor of global E-14 lineages existed in the USA. First, USA was the first country reported to detect E-14 [11] and second, the USA strain is phylogenetically more diverse within the phylogenetic tree (Fig. 2) and the MCC tree (Fig. 3) than those from elsewhere. However, these observations may reflect differences in AFP surveillance intensity among countries and more information is required before we can conclude that E-14 was introduced to the USA multiple times from other regions. Interestingly, our results indicate that circulation of E-14 in Australia is very dynamic, as the virus likely to be the reservoir for evolutionary origin of E-14 in the world. The results that the nucleotide substitution rate of E-14 VP1 gene was similar to other EV serotypes [29–34] also suggested the evolutionary pressure acting on E-14 existed no significant difference with other serotype strains.

A particular focus of this study was to determine the phylogeographic relationship between E-14 collected from Shandong province and compare these with those from other countries. To our knowledge, the current study is the first applying the Bayesian phylogeographic reconstruction approach to gain insight into the emergence and spread of E-14 strains worldwide [35]. The phylogeographic analysis revealed that the transmission of E-14 in China likely originated from the Australia, and then the endemic circulation was observed without spreading to foreign countries. However, E-14 strains in China and Australia have no direct evolutionary relationship according to the phylogenetic analysis. The reason why the phylogenetic and phylogeographic analysis was inconsistent between E-14 strains in China and Australia remains unclear, and analysis of more sequences with greater detail information will yield more precise diffusion process. In addition to spread from Australia to India, E-14 strains seem to have been reintroduced into Australia from India, and the same scene occurred among Shandong province with Guangdong and Yunnan provinces in China. We believe that large-scale patterns in human mobility between these locations will provide a more plausible explanation on E-14 emergence and introduction. Overall, as a well documented origin from the USA, E-14 in this area has become silent over the past few decades. Australia may be responsible for the transmission of E-14 in recent years, and two regional transmissions were mainly detected, with one located between Europe and Africa countries and the other was in the Asia-Pacific region.

The results in this study are subject to a selection bias. Sequences from GenBank are limited, especially for the complete VP1 genes of E-14. In addition, the first E-14 sequence represents a strain isolated in 1954 in the USA, whereas the next sequences can be obtained from GenBank originated in 1980. Therefore, partial VP1 sequences were used for the ananlysis of the phylogenetic relationships among global E-14 strains. Due to availability of limited gene information of global E-14, further collection and analysis, together with detailed epidemiological information is required to understand the spatiotemporal dynamics of E-14 worldwide. All these indicate that EV surveillance in most countries remains to be strengthened and the systematic surveillance are necessary in investigating the EV circulation and understanding the social burden of EV infection.

## Conclusions

In this study, we collected the complete VP1 sequences of E-14 based on the 26-year acute flaccid paralysis surveillance in Shandong province, and inferred the historical background and geographic diffusion of global E-14 strains. The estimated nucleotide substitution rate for global E-14 was 9.17 × 10^−3^ substitutions/site/year, and the estimated *t*_*MRCA*_ was August 1928. Our research confirmed that the origin of E-14 could be in the USA, however, regional transmissions were detected with one located between Europe and Africa countries and the other was in the Asia-Pacific region. Australia may be responsible for the spread of E-14 in recent years.

## Methods

### Virus isolation

Stool samples were collected from children aged <15 years who were presenting acute flaccid type of paralysis and the contacts were collected, and processed according to standard procedures recommended by the WHO [36]. Contacts were defined as high-risk AFP cases with (1) <5 years of age, (2) <3 doses of oral poliovirus vaccine (OPV) immunization or unknown OPV history, (3) inadequate stool specimen or (4) diagnosis of clinical poliomyelitis. The AFP surveillance has been ongoing without any changes since 1990 in line with the WHO recommended standards. Three cell lines, human rhabdomyosarcoma (RD) cell, human epidermoid carcinoma (Hep-2) cell and mouse cell expressing the gene for the human cellular receptor for poliovirus (L20B) were used for virus isolation [36]. All cell lines were gifted from the WHO Global Poliovirus Specialized Laboratory in the USA and were all originally purchased from the American Type Culture Collection. A total of 200μl of chloroform-treated stool solution was added to each of the cell culture tubes, and the inoculated cells were examined daily. After 7 days, the tubes were frozen, thawed, and re-passaged and another 7-day examination was performed. Infected cell cultures were harvested and used for further examination until complete cytopathic effect (CPE) was obtained. To avoid cross contamination, cell tubes of normal L20B, RD and Hep-2 cells served as negative controls. Isolates from RD or Hep-2 cell lines were re-passaged to L20B cell line, and were designated as NPEV if no CPE was observed.

### Nucleotide sequencing and molecular typing

Total RNA was extracted from 140μl of the infected cell culture using QIAamp viral RNA mini kit (Qiagen, Valencia, CA, USA) according to the manufacturers’ recommended procedure. Primer pairs 008/013 [37] and 187/011 [38] were used to amplify VP1 gene and the combination of the two sequences yielded the entire VP1 coding region. The reverse transcription-PCR (RT-PCR) was performed using Access RT-PCR System (Promega, USA) according to the manufacturers’ procedures. In order to avoid cross contamination, a RT-PCR reaction using the RNA extracted from normal RD cell served as a blank control, and a negative control containing all the components of the reaction except for the template was also included.

PCR amplicons were visualized in agarose gels, and positive products were purified and sequenced with the BigDye Terminator v3.0 Cycle Sequencing kit (Applied Biosystems, Foster City, CA). The nucleotide sequences were analyzed by ABI 3130 genetic analyzer (Applied Biosystems, Hitachi, Japan). The PCR products were sequenced in both directions to avoid possible ambiguous nucleotides. Molecular typing based on VP1 sequences was performed using BLAST, obtained online from the National Center for Biotechnology Information (NCBI). Isolates showing >75% nucleotide sequence identities with the E-14 Tow prototype strain were designated relative serotype [37].

### Homologous comparison and phylogenetic analysis

VP1 sequences of E-14 isolated in other countries were blasted and collected by using the nucleotide program in the NCBI. All of the references were downloaded with the strain name, GenBank number, geographical region and collection date. Nucleotide sequence alignments were carried out by BioEdit software v7.0.5.3 [39]. Phylogenetic trees were constructed by using MEGA v6.0 [40], using the neighbor-joining method after estimation of genetic distance using the Kimura two-parameter method [41]. A bootstrapping test was performed with 1,000 duplicates, and the transition/transversion rate was set at 2.0. To the sequences detected in worldwide, we chose one sequence to represent sequences with similarity over 99 percentage and identical isolation years. Recombination can sometimes bias evolutionary relationship when constructing phylogenetic trees [42], hence recombination of VP1 sequences was excluded based on the analysis of SimPlot.

Bayesian Markov Chain Monte Carlo (MCMC) method was used to analyze the evolution rate and molecular clock phylogeny of global E-14 strains containing Shandong isolates with the BEAST software package version 1.7.5 [43], and the time of the most recent common ancestor (*t*_*MRCA*_) with 95% highest posterior density (HPD) of global E-14 and Shandong isolates were estimated (Additional file 3). The calibration point was the year that each strain was isolated. We used the general time reversible (GTR) nucleotide substitution model with gamma-distributed rates of variable among sites, which were identified as the best fitting model by jModelTest v2.1.7 on the basis of Akaike Information Criterion (AIC) [44]. Runs were performed using the constant size, under the strict clock model and the uncorrelated log-normal distributed (UCLD) relaxed molecular clock model. Multiple combinations of molecular clock and coalescent models were explored, and to select the best fitting model we used posterior-simulation Akaike information content (AICM) [45] and Harmonic Mean [46] model selection methods described by Raftery and Baele, respectively. Bayesian MCMC analyses were run with a chain of 30 million steps and sampled every 1,000 steps. Convergence of parameters was identified by TRACER v1.5 with the effective sample size (ESS) exceeding 200. The Maximum Clade Credibility (MCC) tree was calculated by TreeAnnotator with a burn-in period of 3,000 and then visualized in FigTree v1.4.2.

### Phylogeographic analysis

In order to infer the spatiotemporal dynamics of E-14, the Bayesian Stochastic Search Variable Selection (BSSVS) was used to provide evidence for statistically supported diffusion between state variables under BEAST v1.7.5. The results of BSSVS was summarized using SPREAD v1.0.6 [47], a keyhole markup language (KML) file was generated to identify the major routes of geographic diffusion (Additional file 4). Bayes factor (BF) test was used to select the most probable diffusion process. To visualize the geographic dispersal of E-14, the KML file was imported to Google™ Earth to produce a graphical animation of the estimated spatiotemporal pathways of global E-14.

### Nucleotide sequence accession numbers

The VP1 sequences of E-14 isolates described in this study were deposited in the GenBank database under accession numbers KX840391-KX840454. All sequences data that support the findings of this study have been deposited in TreeBASE and are accessible through the accession number 20306 (http://purl.org/phylo/treebase/phylows/study/TB2:S20306).

## Additional files

Additional file 1: Geographic distribution and inferred dynamics of E-14 created by the SPREAD software. (TIF 6150 kb)
Additional file 2: A graphical animation of the estimated spatiotemporal pathways of global E-14 produced by Google™ Earth. (PDF 2809 kb)
Additional file 3: The XML file generated for the phylogenetic analysis with BEAST software. (XML 105 kb)
Additional file 4: The KML file generated for the spatiotemporal analysis of global E-14 in this study. (KML 2866 kb)

## Abbreviations

AFP: Acute flaccid paralysis
AIC: Akaike information criterion
AICM: Akaike information content
BF: Bayes factor
BSSVS: Bayesian stochastic search variable selection
CPE: Cytopathic effect
CV: Coxsackieviruses
E-14: Echovirus 14
EG: Exponential growth
ESS: Effective sample size
EVs: Enteroviruses
GTR: General time reversible
Hep-2: Human epidermoid carcinoma
HFMD: Hand, foot and mouth disease
HPD: Highest posterior density
KML: Keyhole markup language
L20B: Mouse cell expressing the gene for the human cellular receptor for poliovirus
MCC: Maximum Clade Credibility
MCMC: Bayesian Markov Chain Monte Carlo
NCBI: National Center for Biotechnology Information
NPEV: Non-polio enterovirus
OPV: Oral poliovirus vaccine
RD: Human rhabdomyosarcoma
RT-PCR: Reverse transcription-PCR
*t*_*MRCA*_: The time of the most recent common ancestor
UCLD: Uncorrelated log-normal distributed
