# Supplementary figures and images for: Evolutionary phylogeography and transmission pattern of echovirus 14: an exploration of spatiotemporal dynamics based on the 26-year acute flaccid paralysis surveillance in Shandong, China
Source: BMC Genomics. 2017 Jan 7;18:48. doi: 10.1186/s12864-016-3418-3 (PMC5219651; doi:10.1186/s12864-016-3418-3)

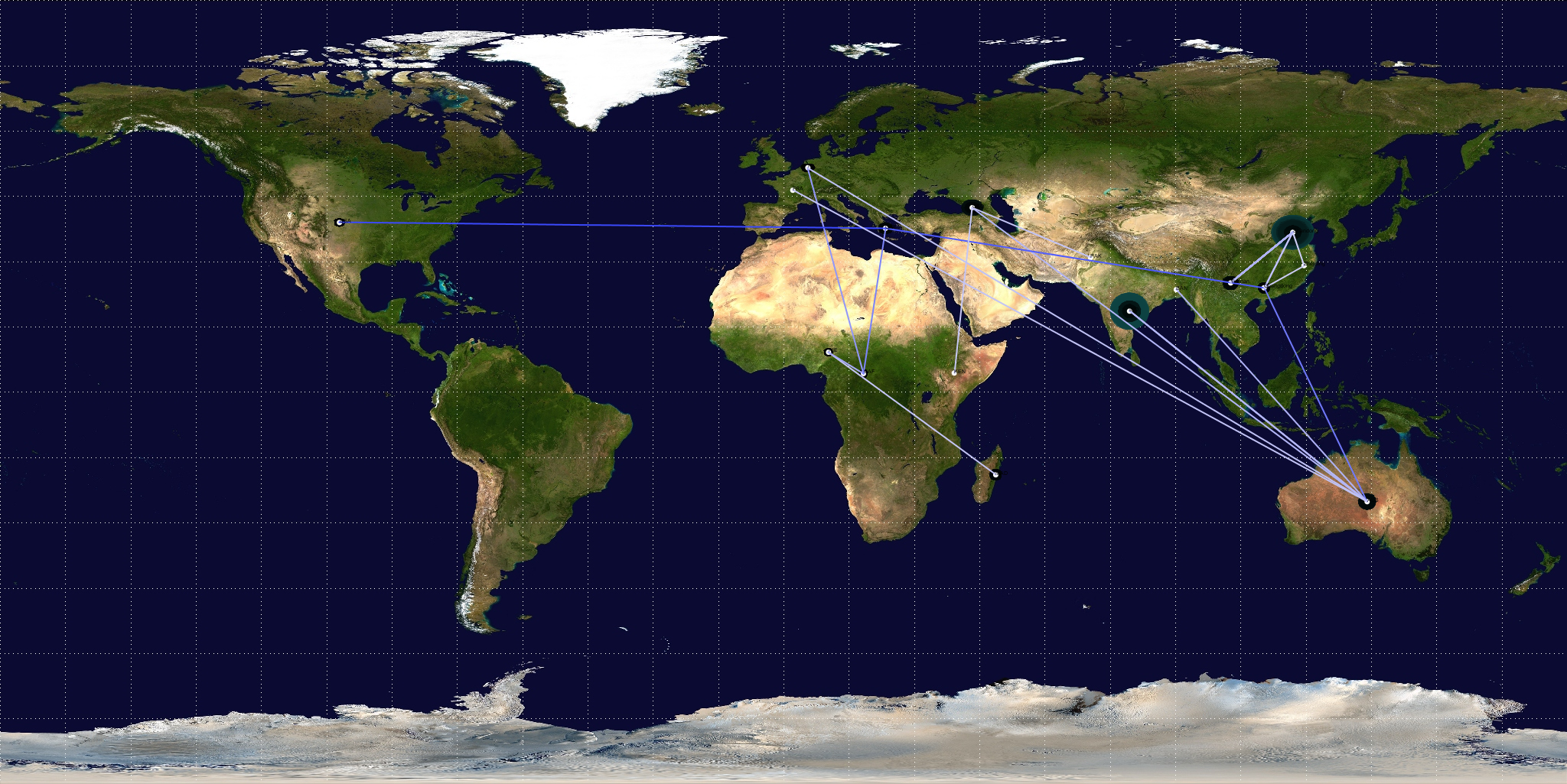

Supplement: Additional file 1: — Geographic distribution and inferred dynamics of E-14 created by the SPREAD software. (TIF 6150 kb) [file 12864_2016_3418_MOESM1_ESM.tif]

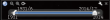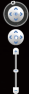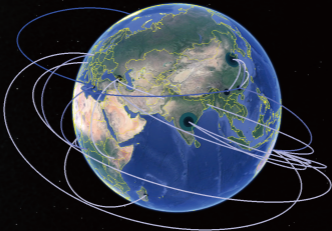

U.S. Dept. of State Geography  
Data SIO, NOAA, U.S. Navy, NGS, GEBCO  
© 2005 Google  
Image Landsat

Google earth

Supplement: Additional file 2: — A graphical animation of the estimated spatiotemporal pathways of global E-14 produced by Google™ Earth. (PDF 2809 kb) [file 12864_2016_3418_MOESM2_ESM.pdf]
